# P-1962. Risk Factors, Clinical Outcomes and Immune Dynamics of Candidemia in Patients with Hematological Disorder

**DOI:** 10.1093/ofid/ofaf695.2129

**Published:** 2026-01-11

**Authors:** Zhangjie Chen, Sisi Zhen, Xin Chen, Sizhou Feng

**Affiliations:** Institute of Hematology & Blood Diseases Hospital, Chinese Academy of Medical Sciences & Peking Union Medical College, Tianjin, Tianjin, China (People's Republic); Institute of Hematology & Blood Diseases Hospital, Chinese Academy of Medical Sciences & Peking Union Medical College, Tianjin, Tianjin, China (People's Republic); Institute of Hematology & Blood Diseases Hospital, Chinese Academy of Medical Sciences & Peking Union Medical College, Tianjin, Tianjin, China (People's Republic); Institute of Hematology & Blood Diseases Hospital, Chinese Academy of Medical Sciences & Peking Union Medical College, Tianjin, Tianjin, China (People's Republic)

## Abstract

**Background:**

Candidemia, particularly due to *Candida tropicalis*, was of high incidence among patients with hematological disorders. However, associated risk factors, immune dynamics and clinical outcomes remained underexplored.

**Figure 1:**
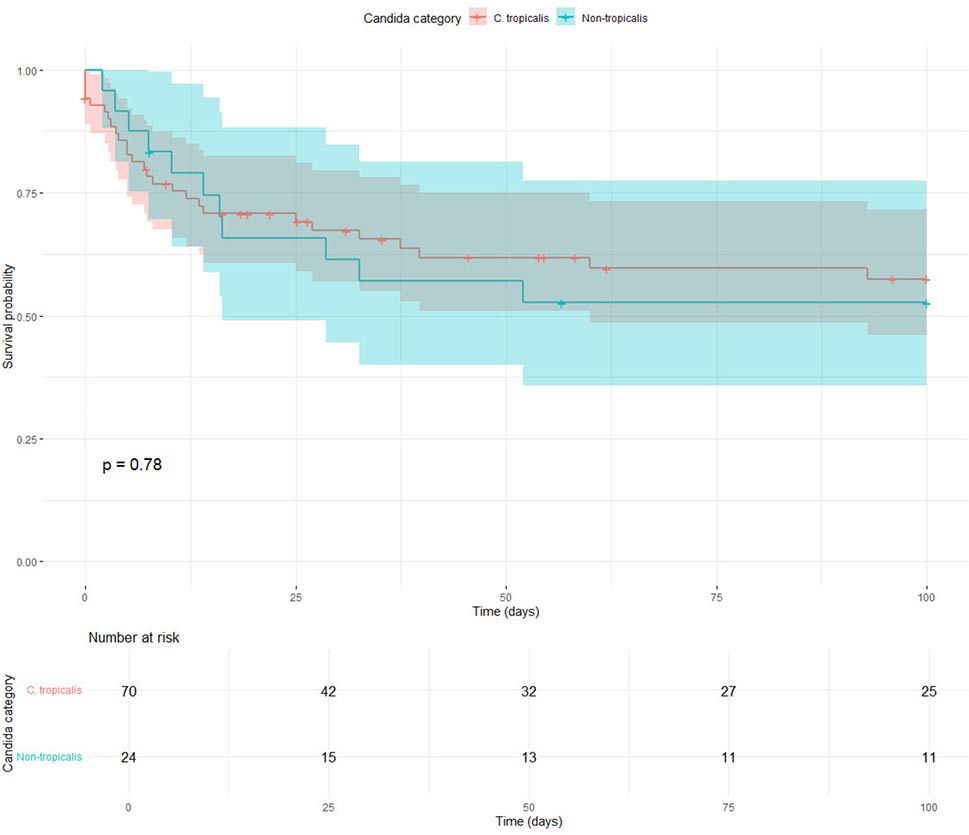
Survival analysis of patients with Candida tropicalis versus non-tropicalis candidemia (C).

**Figure 2:**
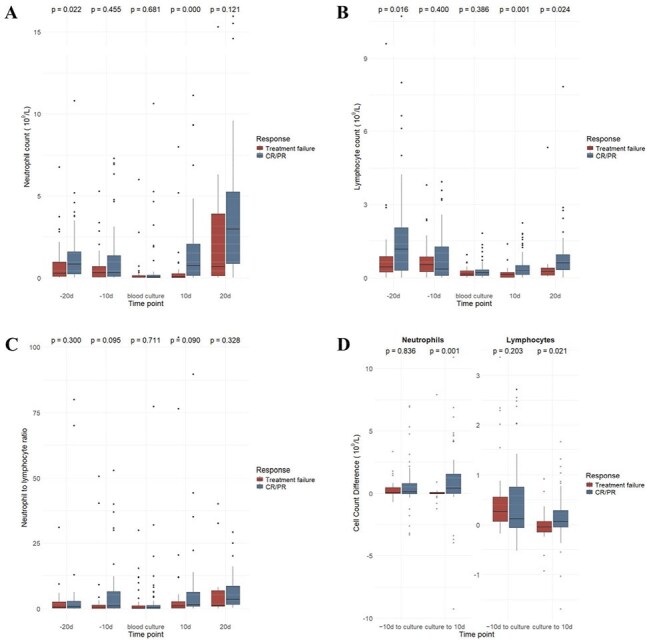
Neutrophil count (A), lymphocyte count (B), neutrophil to lymphocyte ratio (C) and changes in neutrophil and lymphocyte count (D) at multiple time points in patients with complete response (CR)/partial response (PR) or treatment failure.

**Methods:**

Clinical data from 105 patients diagnosed with hematological disorders and confirmed candidemia were retrospectively analyzed.

**Figure 3:**
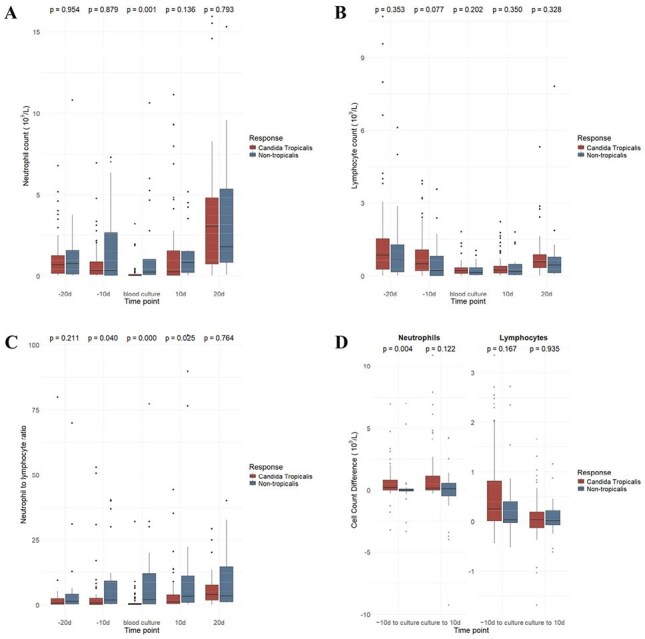
Neutrophil count (A), lymphocyte count (B), neutrophil-to-lymphocyte ratio (C) and changes in neutrophil and lymphocyte count (D) at multiple time points in patients with Candida tropicalis or non-tropicalis candidemia.

**Results:**

*Candida tropicalis* was identified in 79 patients (75.2%), and non-tropicalis species included Candida albicans (n=6, 5.7%), Candida glabrata (n=4, 3.8%), Candida parapsilosis (n=12, 11.4%), Candida krusei (n=3, 2.9%) and C. guilliermondii (n=1, 1.0%). Prior transplantation was more common in non-tropicalis candidemia (P=0.023), whereas culture positivity within 24 hours was associated with *Candida tropicalis* (P=0.008). *Candida tropicalis* showed favorable antifungal susceptibility compared to non-tropicalis, particularly to flucytosine (P< 0.001). Azole monotherapy (29.5%) and azole-echinocandin combination (25.7%) were the most used treatments. Complete response was achieved in 58 patients (55.2%), with similar rate between *Candida tropicalis* and non-tropicalis group (P = 0.869) (Figure 1). The restricted median survival time was 64 days, with no significant difference in mortality or survival between groups. Older age (P=0.043), abdominal pain (P=0.040) and septic shock (P=0.008) were associated with poor outcomes. Patients achieving remission exhibited higher neutrophil and lymphocyte counts and greater immune recovery (Figure 2). *Candida tropicalis* BSI was associated with deeper neutropenia, significantly lower NLR and a more pronounced neutrophil decline at onset (Figure 3), suggesting a “double-hit” immune suppression.

**Conclusion:**

*Candida tropicalis* and non-tropicalis candidemia exhibited distinct risk factors and immune dynamics, yet both were associated with high mortality. Further investigation is needed to elucidate invasion mechanism and immunological vulnerabilities, guiding targeted prophylaxis and therapy.

**Disclosures:**

All Authors: No reported disclosures

